# A new species and additional records of *Rugilus* Leach from Qinling, China (Coleoptera, Staphylinidae, Paederinae)

**DOI:** 10.3897/zookeys.505.9681

**Published:** 2015-05-25

**Authors:** Jia-Yao Hu, Chen-Zu Song, Li-Zhen Li

**Affiliations:** 1 Department of Biology, College of Life and Environmental Sciences, Shanghai Normal University, 100 Guilin Road, Shanghai, 200234 P. R. China

**Keywords:** Coleoptera, Staphylinidae, Paederinae, Rugilus, Qinling, China, new species

## Abstract

A new species of *Rugilus* Leach, Rugilus (Rugilus) huanghaoi **sp. n.** from Qinling, Shaanxi Province, China, is described and illustrated. Additional records of seven species from Qinling are reported.

## Introduction

According to a series of revisions of the genus *Rugilus* Leach from Palaearctic and Oriental regions (Assing 2012a, 2012b, 2013, 2014, 2015), 32 species of the genus have been recorded from China, 25 of them are placed in the nominotypical subgenus, and seven in the subgenus *Eurystilicus* Fagel. Seven species have been report fromed the Qinling Shan, one of the most diverse areas in China: Rugilus (Eurystilicus) rufescens (Fauvel, 1874), Rugilus (Eurystilicus) simlaensis (Cameron, 1931), Rugilus (Eurystilicus) velutinus (Fauvel, 1895), Rugilus (Rugilus) dabaicus Assing, 2012, Rugilus (Rugilus) fodens Assing, 2012, Rugilus (Rugilus) gansuensis Rougemont, 1998, and Rugilus (Rugilus) reticulatus Assing, 2012. During several recent field trips to this region conducted by the authors and their colleagues, all known and an additional new species were collected.

## Material and methods

The type material listed in the present study is deposited in the Insect Collection of Shanghai Normal University, Shanghai, P. R. China (SNUC).

The dissected body parts were mounted in Euparal on plastic slides. The habitus photos were taken using a Canon 7D camera. The photos of the sternites and aedeagi were taken using a Canon G9 camera mounted on an Olympus CX31 microscope.

### Measurements:

Body length: measured from the anterior margin of the labrum to the apex of the abdomen.

Length of forebody: measured from anterior margin of the labrum to the posterior margin of the elytra.

Eye length: longitudinal length of eye in dorsal view.

Postocular length: measured from posterior margin of eye to posterior constriction of head.

Head width: width of head across (and including) eyes.

Head length: measured from the clypeal anterior margin to head base.

Pronotum width: maximal width of pronotum.

Pronotum length: measured in midline from front margin to posterior margin.

Width of elytra: combined width of elytra at posterior margin.

Length of elytra: measured from apex of scutellum to posterior margin.

## Description of new species

### Rugilus (Rugilus) huanghaoi sp. n.

Taxon classificationAnimaliaColeopteraStaphylinidae

http://zoobank.org/8E11E667-B5DC-4D85-A334-153047FE5BDC

Figs 1–7

#### Type material.

Holotype: male: “China: Shaanxi Prov., Zhouzhi County, Houzhenzi, Qinling, Qinlingliang, N33.48.963, E107.44.483, alt. 2018 m, 7.V.2008, HUANG Hao & XU Wang leg.” (SNUC). Paratypes: 1 male, 1 female: “China: Shaanxi Prov., Mei County, Taibai Shan, Kaitianguan, N34.00.692, E107.51.415, alt. 1853 m, 22–23.V.2008, HUANG Hao & XU Wang leg.”.

#### Description.

Body length 5.4–6.4 mm; forebody length: 4.1–4.3 mm.

**Figures 1–7.**
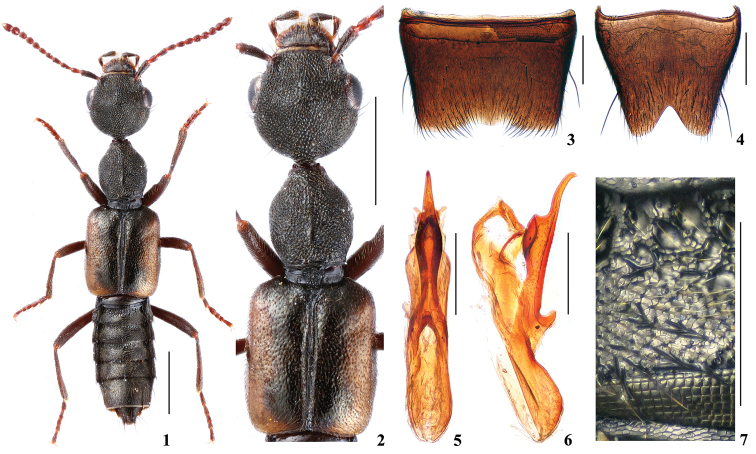
Rugilus (Rugilus) huanghaoi sp. n. **1** habitus **2** forebody **3** male sternite VII **4** male sternite VIII **5** aedeagus in ventral view **6** aedeagus in lateral view **7** median portion of tergite III. Scale bars: 1 mm (**1, 2**), 0.25 mm (**3–7**).

Body (Fig. 1) dark brown; lateral margins of elytra widely yellowish brown; legs and antennae reddish brown.

Head (Fig. 2) 0.96–0.97 times as long as wide; punctation umbilicate, very dense, rather coarse and partly confluent, interstices reduced to very narrow ridges; without microsculpture. Eyes large and convex; approximately 0.8–0.9 times as long as postocular portion. Anterior margin of labrum with two pronounced teeth on either side of the median incision.

Pronotum (Fig. 2) 1.16–1.17 times as long as wide, 0.75–0.77 times as broad and 0.92–0.94 times as long as head; punctation similar to that of head; midline with narrow and short impunctate elevation in posterior half; interstices without microsculpture. Elytra (Fig. 2) 0.90–0.98 times as long as wide, 1.13–1.26 times as long and 1.47–1.52 times as broad as pronotum; punctation dense, distinctly finer than that of head and pronotum; interstices without microsculpture.

Abdomen narrower than elytra; tergites III-VI with transverse impressions anteriorly. Punctation of these impressions coarse and dense; punctation of remaining surfaces fine and dense; interstices with distinct microsculpture (Fig. 7); posterior margin of tergite VII with distinct palisade fringe.

*Male*. Sternite VII (Fig. 3) with broad and trapezoidal excision in the middle of posterior margin; on either side of this excision with a tuft of long black setae. Sternite VIII (Fig. 4) with triangular excision posteriorly. Aedeagus (Figs 5, 6) long and narrow; ventral process widest near middle and gradually narrowed apically in ventral view; abruptly narrowed and slightly curved ventrally in apical third in lateral view.

#### Comparative notes.

Based on the similar external characters, especially the bicoloured elytra and the male sexual characters, the new species is most similar to *Rugilus morvani* (Rougemont, 1987) from Nepal, from which it is distinguished by the deeper excision of the male sternite VII, with a more prominently produced centre, longer and denser setae on either side of the excision, and by the longer and narrower apical portion of the aedeagal ventral process.

#### Distribution and habitat data.

The species was found in two localities in the Qinling Shan. The specimens were collected by sifting decaying leaf litter in mixed forests at altitudes from ca. 1850 to 2020 m. The paratypes were collected together with *Rugilus reticulatus*.

#### Etymology.

The species is named in honor of Hao Huang, one of the collectors of the type material.

### New records

#### Rugilus (Eurystilicus) rufescens

Taxon classificationAnimaliaColeopteraStaphylinidae

(Fauvel, 1874)

##### Material examined.

**CHINA: Shaanxi:** 2 males, Foping, 850–950 m, 20.VII.2004, Hu, Tang & Zhu leg.

##### Comment.

The species is widespread in the East Palaearctic and Oriental regions (Assing 2012a).

#### Rugilus (Eurystilicus) simlaensis

Taxon classificationAnimaliaColeopteraStaphylinidae

(Cameron, 1931)

##### Material examined.

**CHINA: Shaanxi:** 1 female, Ankang City, Ningshaan County, Huoditang Foresty Centre, 33°26'N, 108°27'E, 1500–1700 m, 12.VII.2012, Li-Zhen Li leg.

##### Comment.

The distribution of this species ranges from the Himalaya to Mainland China and Taiwan (Assing 2012a). The above material was collected together with *Rugilus velutinus*.

#### Rugilus (Eurystilicus) velutinus

Taxon classificationAnimaliaColeopteraStaphylinidae

(Fauvel, 1895)

##### Material examined.

**CHINA: Shaanxi:** 3 males, Ankang City, Ningshaan County, Huoditang Foresty Centre, 33°26'N, 108°27'E, 1500–1700 m, 12.VII.2012, Li-Zhen Li leg.; 1 female, Hanzhong City, Nanzheng County, Yuanba Town, Liping National Forest Park, 32°50'N, 106°36'E, 1400–1600 m, 16.VII.2012, Yu-Hong Pan leg.

##### Comment.

The species is widespread in the East Palaearctic and Oriental regions (Assing 2012a). Some of the above material was collected together with *Rugilus simlaensis*.

#### Rugilus (Rugilus) dabaicus

Taxon classificationAnimaliaColeopteraStaphylinidae

Assing, 2012

##### Material examined.

**CHINA: Shaanxi:** 5 males, 16 females, Ningshaan County, Qinling, Huoditang Linchang, N33.26.060, E108.26.291, 1724 m, 24–25.V.2008, Hao Huang & Wang Xu leg.; 19 females, same locality, 1500–1700 m, 12.VII.2012, Yan Chen, Li-Zhen Li, Wen-Rong Li, Wen-Li Ma, Yu-Hong Pan & Jie-Qiong Zhao leg.; 15 females, Foping, 1250–1400 m, 18.VII.2004, Hu, Tang & Zhu leg.

##### Comment.

The species was originally described from Daba Shan in Hubei (Assing 2012a) and recently recorded from Qinling Shan in Shaanxi (Assing 2015).

#### Rugilus (Rugilus) fodens

Taxon classificationAnimaliaColeopteraStaphylinidae

Assing, 2012

##### Material examined.

**CHINA: Shaanxi:** 2 males, 5 females, Hanzhong City, Nanzheng County, Yuanba Town, Liping National Forest Park, 32°50'N, 106°36'E, 1400–1600 m, 15.VII.2012, Chen, Li, Ma & Zhao leg.; 2 males, 2 females, same locality, 16.VII.2012.

##### Comment.

This species was previously known from Daba Shan in Hubei and Micang Shan in Sichuan and Shaanxi (Assing 2012a). The above material was collected together with *Rugilus gansuensis*.

#### Rugilus (Rugilus) gansuensis

Taxon classificationAnimaliaColeopteraStaphylinidae

Rougemont, 1998

##### Material examined.

**CHINA: Shaanxi:** 1 male, 2 females, Zhouzhi County, Houzhenzi, Qinling, West Sangongli Gou, N33.50.613, E107.48.524, 1336 m, 17–19.V.2008, Hao Huang & Wang Xu leg.; 1 male, Zhouzhi County, Houzhenzi, Qinling, N33.51.203, E107.50.183, 1260 m, 5–10.V.2008, Hao Huang & Wang Xu leg.; 1 male, 2 females, Hanzhong City, Nanzheng County, Yuanba Town, Liping National Forest Park, 32°50'N, 106°36'E, 1400–1600 m, 15.VII.2012, Chen, Li, Ma, Pan & Zhao leg.

##### Comment.

This species is widespread in Qinling from Gansu to Shaanxi (Assing 2012a, 2013). Some of the above material was collected together with *Rugilus fodens*.

#### Rugilus (Rugilus) reticulatus

Taxon classificationAnimaliaColeopteraStaphylinidae

Assing, 2012

##### Material examined.

**CHINA: Shaanxi:** 2 males, 10 females, Zhouzhi County, Qinling, Daoban, N38.43.645, E107.58.147, 1900 m, 4.V.2008, Hao Huang & Wang Xu leg.; 1 male, Mei County Taibai Shan, Kaitianguan, N34.00.692, E107.51.415, 1853 m, 22–23.V.2008, Hao Huang & Wang Xu leg.; 11 females, Mt. Taibai, 1450–1750 m, 15.VII.2004, Hu & Tang leg.; 3 females, Foping, 2065 m, 21.VII.2004, Hu, Tang & Zhu leg.

##### Comment.

The species was known from Qinling in Shaanxi and Funiu Shan in Henan (Assing 2012a). Some of the above material was collected together with *Rugilus huanghaoi*.

## Supplementary Material

XML Treatment for Rugilus (Rugilus) huanghaoi

XML Treatment for Rugilus (Eurystilicus) rufescens

XML Treatment for Rugilus (Eurystilicus) simlaensis

XML Treatment for Rugilus (Eurystilicus) velutinus

XML Treatment for Rugilus (Rugilus) dabaicus

XML Treatment for Rugilus (Rugilus) fodens

XML Treatment for Rugilus (Rugilus) gansuensis

XML Treatment for Rugilus (Rugilus) reticulatus
